# Liver Cirrhosis Secondary to Autoimmune Hepatitis in a Patient with Alpha-1 Antitrypsin ZZ Phenotype: A "Double Hit" Phenomenon

**DOI:** 10.7759/cureus.12606

**Published:** 2021-01-10

**Authors:** Gilles J Hoilat, Ayesha Khan, Umair Masood, Anuj Sharma, Divey Manocha

**Affiliations:** 1 Internal Medicine, Upstate Medical University, State University of New York, Syracuse, USA; 2 Internal Medicine, American University of Integrative Sciences, Cole Bay, BRB; 3 Gastroenterology, Upstate Medical University, State University of New York, Syracuse, USA

**Keywords:** live cirrhosis, liver biopsy, autoimmune cirrhosis, alpha-1 antitrypsin

## Abstract

Alpha-1 antitrypsin deficiency has been known to cause pulmonary and hepatic diseases. Cirrhosis in patients with alpha-1 antitrypsin deficiency, especially in a homozygotes ZZ phenotype, has been described to occur exclusively as a congenital disease. We present the case of a young 28-year-old female who was initially followed for thrombocytopenia and was found to have cirrhosis of the liver with autoimmune histological features suggesting the possibility that another “second hit” can contribute to a more rapid progression of liver disease.

## Introduction

Alpha-1 antitrypsin (AAT) deficiency is a proteinopathy, a disease caused by the abnormal synthesis, folding, post-translational modification, or deposition of protein in cells or tissues, resulting in impaired cellular function by aggregation of misfolded protein. In the liver, these misfolded proteins accumulate and can lead to hepatic fibrosis and even hepatocellular carcinoma [1]. Although severe AAT deficiency is considered a significant risk factor for the development of cirrhosis in adulthood, there is limited information on other risk factors contributing to it. We present the case of a young woman with an AAT deficiency who developed cirrhosis secondary to an autoimmune phenomenon.

## Case presentation

**Figure 1:**
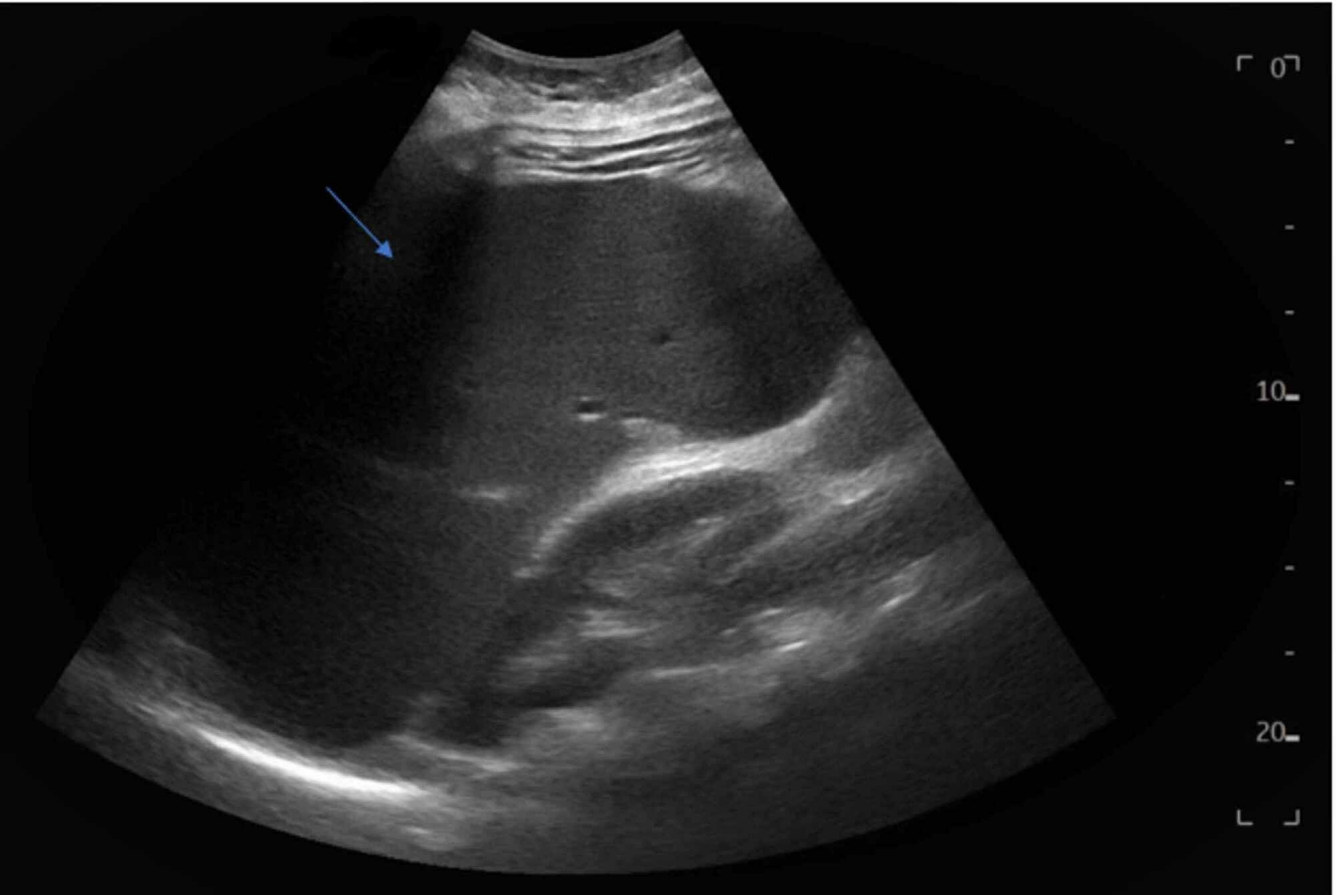
Abdominal ultrasound showing splenomegaly (arrowhead)

**Figure 2:**
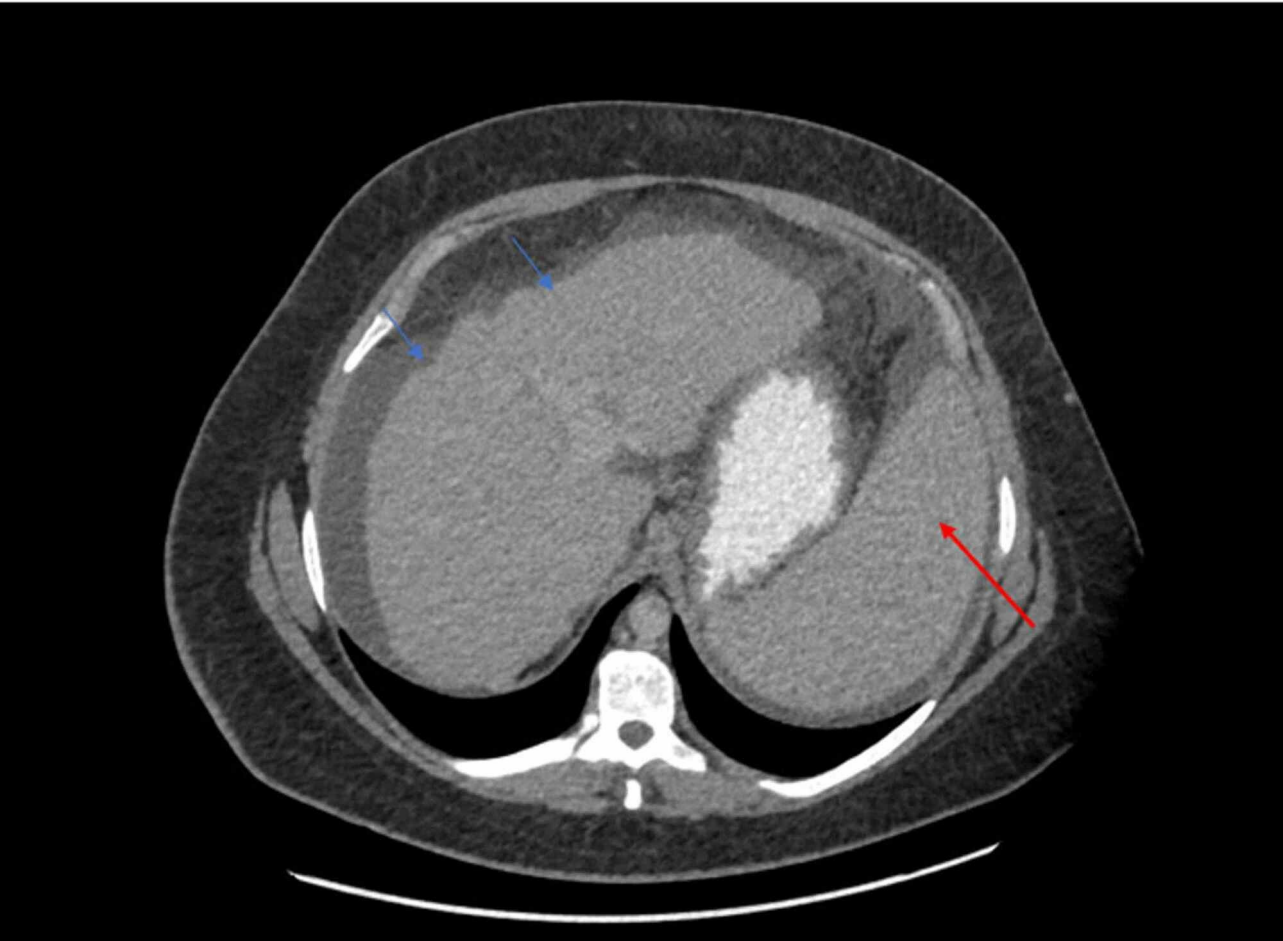
Abdominal computed tomography (CT) showing a nodular contour of the liver concerning for liver cirrhosis (blue arrowhead) and splenomegaly (red arrowhead)

We present the case of a 28-year-old female with no significant past medical history who had been following with the hematology service for isolated thrombocytopenia of 48 10^3^/uL. She underwent a bone marrow biopsy showing thrombocytopenia with slightly increased marrow megakaryocytes, consistent with peripheral destruction of the platelets, and was diagnosed with immune thrombocytopenic purpura. She subsequently underwent an abdominal ultrasound and computed tomography (CT) scan to assess the spleen size and was found to have splenomegaly, moderate abdominal ascites, and a nodular liver (Figures 1-2). She was subsequently referred to the gastroenterology service for management of liver cirrhosis.

**Figure 3:**
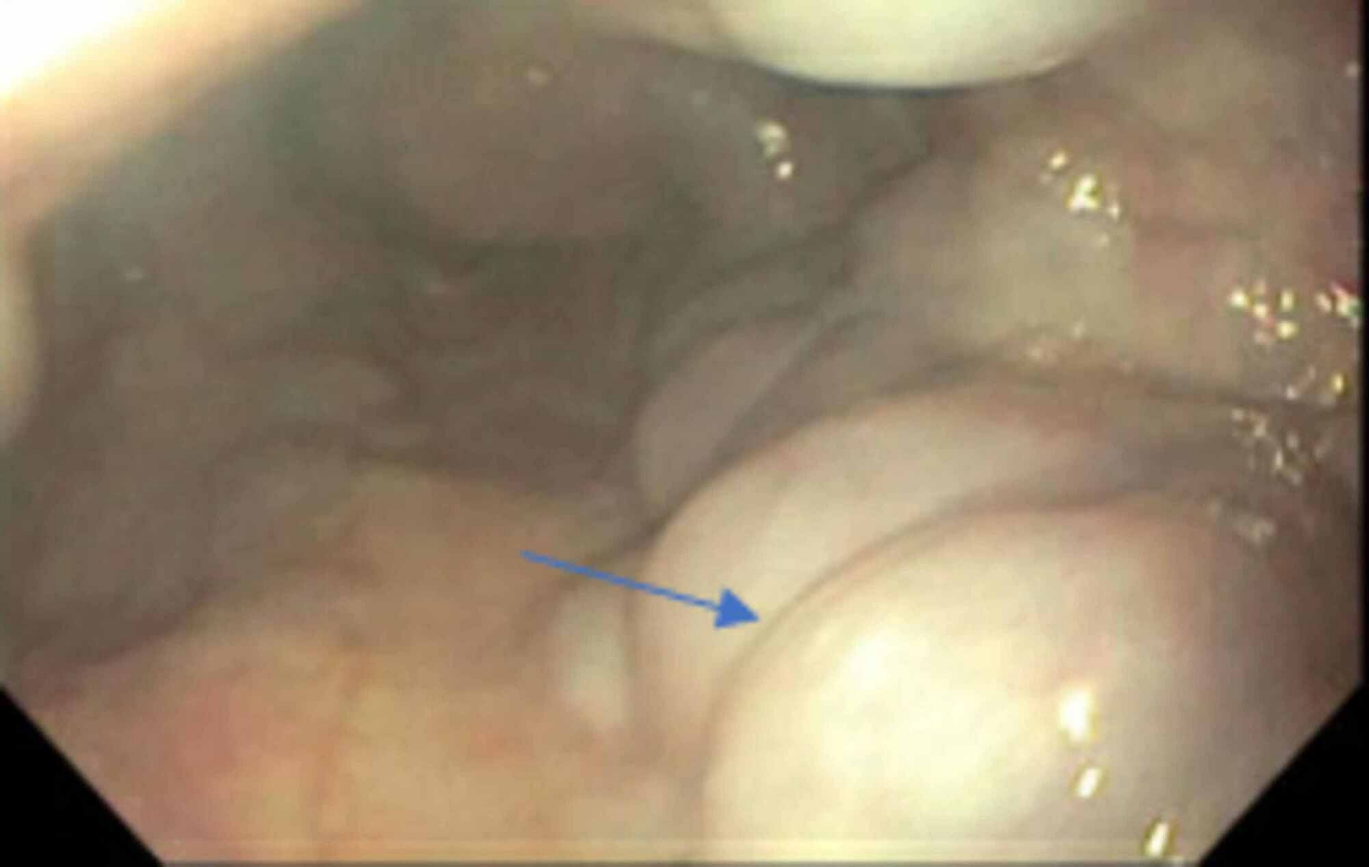
Esophagogastroduodenoscopy (EGD) showing grade II esophageal varices (arrowhead)

**Figure 4:**
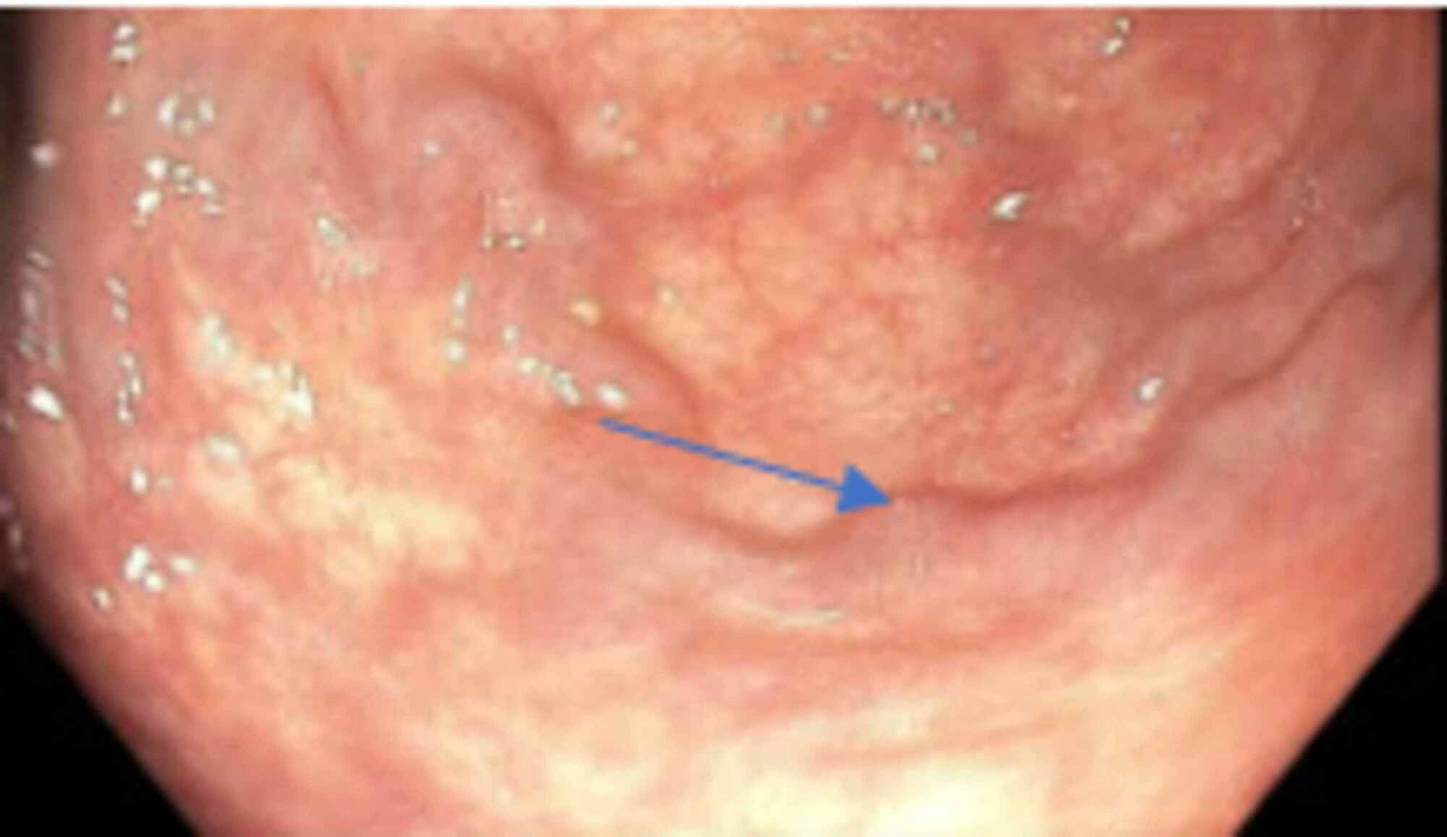
Colonoscopy showing rectal varices (arrowhead)

Liver function tests (LFTs) showed an alanine aminotransferase (ALT) of 41 U/L, aspartate aminotransferase (AST) of 64 U/L, alkaline phosphatase (ALP) of 81 U/L, direct bilirubin of 0.3 mg/dL, and total bilirubin of 1.4 mg/dL. Her model for end-stage liver disease-sodium (MELD-Na) was 15. A complete cirrhosis workup was performed and revealed a negative hepatitis panel, ceruloplasmin levels within normal limits, iron level, total iron-binding capacity, ferritin within normal limit, and a negative anti-mitochondrial antibody. On the other hand, cirrhosis workup was significant for a low AAT level below 20 mg/dL (reference range: 100 - 188 mg/dL) with a ZZ phenotype. Anti-smooth muscle antibody was negative; however, ANA (antinuclear antibody) speckled pattern was elevated to 1:160. Subsequently, immunoglobulin (Ig) levels showed an elevated IgA of 456 mg/dL, an elevated IgG subclass 1 of 1,101 mg/dL, IgG subclass 2 of 511 mg/dL, IgG subclass 3 of 103 mg/dL, and IgG subclass 4 of 224 mg/dL. The patient was started on 50 mg of spironolactone and 20 mg of furosemide for the ascites and underwent an upper endoscopy, as well as a colonoscopy, which were remarkable for Grade II esophageal varices and rectal varices (Figures 3-4). Given the uncertainty of the etiology of her cirrhosis, the decision was made to proceed with a liver biopsy which showed findings of cirrhosis consistent with autoimmune hepatitis with moderate activity.

## Discussion

Alpha-1 antitrypsin deficiency is an inherited genetic disease affecting the lungs and the liver. In the liver, AAT deficiency can present as benign neonatal hepatitis syndrome; few adults develop liver fibrosis, with progression to cirrhosis and hepatocellular carcinoma [2]. AAT is a protease inhibitor that is encoded by the SERPINA1 gene. M is the normal allele while Z refers to the mutated allele.

**Figure 5:**
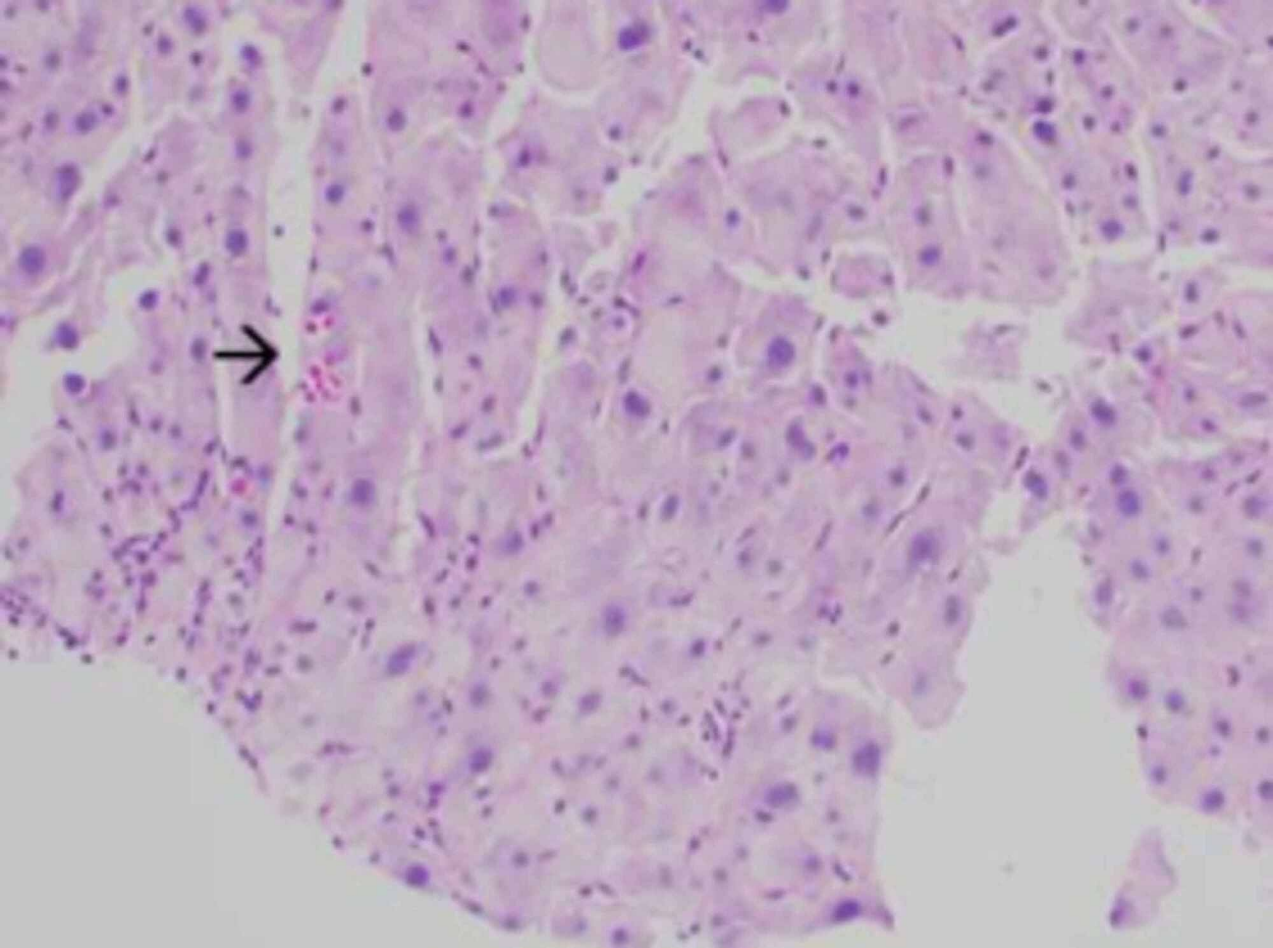
Higher power of hematoxylin and eosin (H&E) sections shows that the areas of severe inflammation (arrowhead) are present at the interface of liver parenchyma with the fibrotic bands. This interface hepatitis is more often seen in autoimmune hepatitis rather than alpha-1 antitrypsin deficiency.

**Figure 6:**
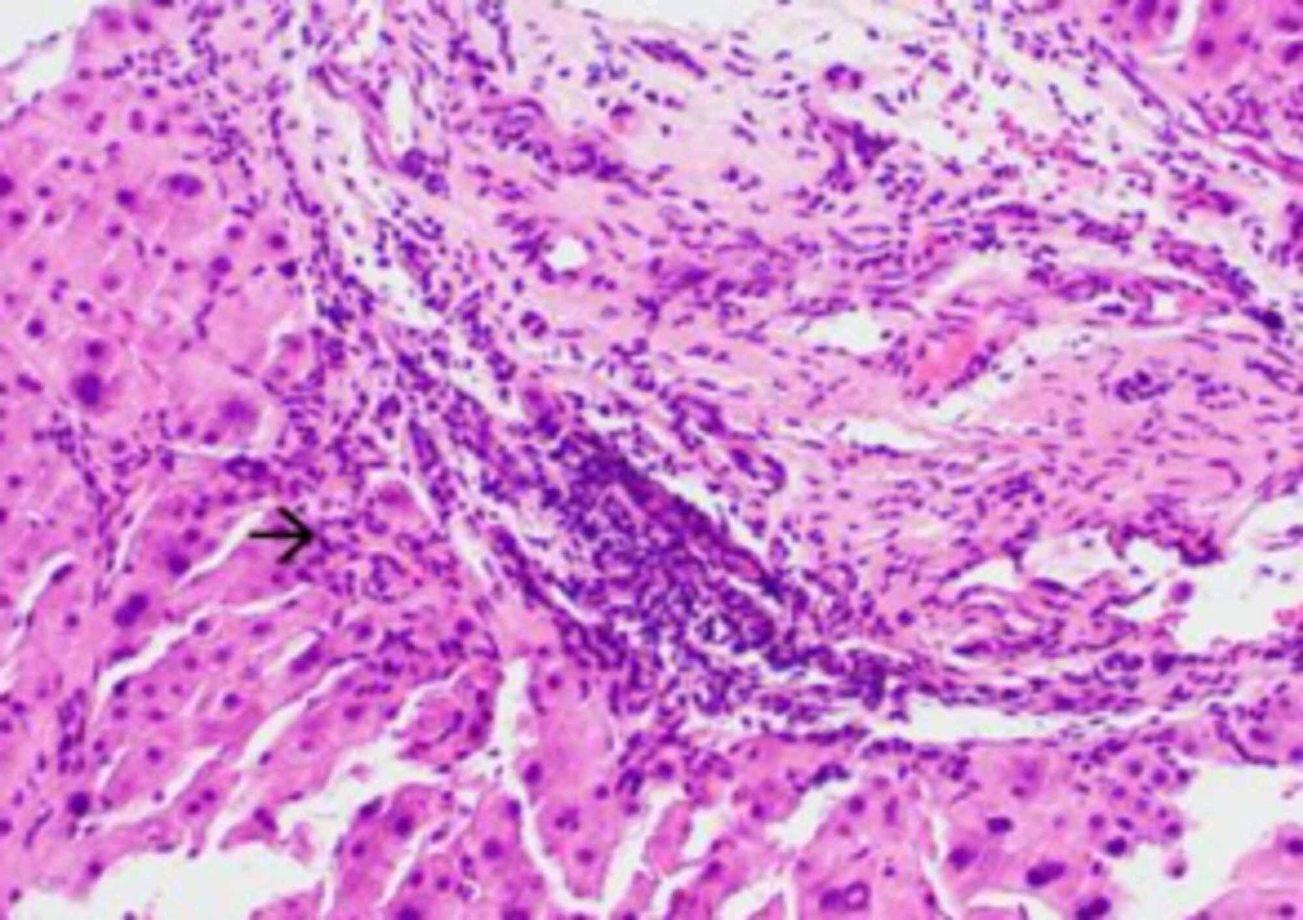
The periodic acid-Schiff (PAS) stain highlights a patchy distribution of intracytoplasmic hyaline globules (arrowhead). These hyaline globules would be expected in a much more diffuse pattern in cirrhosis caused by alpha-1 antitrypsin deficiency.

In homozygous ZZ AAT deficiency, the liver generates large quantities of AAT mutant Z, which folds improperly and is retained within the hepatocytes. These polymers activate an injury cascade, leading to a broad spectrum of liver injury [3]. The homozygous ZZ phenotype typically causes severe liver injury leading to cirrhosis and is characterized histologically by periodic acid-Schiff (PAS)-positive diastase-resistant globules in hepatocyte cytoplasm consistent with retained AAT molecules [4-5]. In our case, histopathological slides were reviewed by two independent pathologists in two different institutions and revealed that the findings of cirrhosis in this patient are more in favor of an autoimmune process than AAT deficiency. Figure 5 reflecting a higher power of hematoxylin and eosin sections shows that the areas of severe inflammation are present at the interface of liver parenchyma with the fibrotic bands. This interface hepatitis is more often seen in autoimmune hepatitis, rather than AAT deficiency. Figure 6, a PAS stain, highlights a patchy distribution of intracytoplasmic hyaline globules. These hyaline globules would be expected in a much more diffuse pattern in cirrhosis caused by an AAT deficiency.

Liver disease secondary to other etiologies has been described in patients with AAT with a heterozygous phenotype. Cirrhosis in AAT deficiency with homozygous ZZ phenotype is almost exclusively due to the inherited condition. This unique case highlights the possibility that another “second hit” insult can contribute to a more rapid progression of liver disease.

## Conclusions

Immune thrombocytopenic purpura is a diagnosis of exclusion and is usually the attributed diagnosis in a healthy young patient with thrombocytopenia. It is important to keep in mind that thrombocytopenia may result from liver dysfunction, and clinicians should maintain a high level of suspicion in an otherwise healthy young thrombocytopenic patient. AAT ZZ phenotype is a rare disease that has been known to cause cirrhosis of the liver in adulthood due to the accumulation of protein in the liver. Autoimmune cirrhosis superimposed on cirrhosis due to AAT is very rare, and this might suggest the possibility that another “second hit” can contribute to a more rapid progression of liver disease.
